# The impact of co‐location employment partnerships within the Australian mental health service and policy context: A systematic review

**DOI:** 10.1111/inm.13007

**Published:** 2022-04-16

**Authors:** Sue Mallick, Md Shahidul Islam

**Affiliations:** ^1^ Vocational Consultant/Senior Occupational Therapist Western Sydney Local Health District (WSLHD) Mental Health Services NSW Health Westmead New South Wales Australia; ^2^ School of Health Faculty of Medicine and Health University of New England Armidale New South Wales Australia

**Keywords:** co‐location, disability employment services, mental illness, policy

## Abstract

Adults with a serious persistent mental illness (SPMI) express a strong desire to work. However, they continue to experience higher levels of unemployment, barriers, and occupational exclusion that impact their vocational outcomes and choice of work. The aim of this study was to investigate the impact of co‐location partnerships between adult mental health and disability employment services (DES) on employment outcomes and consumer choice of work for adults with a SPMI. Following the Preferred Reporting Items for Systematic Reviews and Meta‐Analyses (PRISMA) methodology, a systematic literature review was conducted by searching four databases. The relationship between mental health, employment, and DES was examined. Inclusion criteria were adults with a SPMI; employment services and outcomes; and job retention and sustainability. Twelve studies met inclusion criteria. All studies were peer‐reviewed, Australian‐based, and published between 01 January 2017 and 30 August 2021. Individual placement and support (IPS); DES practice, funding, policy, and reform within the Australian mental health system; and barriers to participation in DES were the three main themes that emerged. Findings highlight the importance of joint, co‐location partnerships between mental health and employment services, including a collaborative approach to policy reform between both services, to assist adults with a SPMI to gain and sustain competitive employment. Vocational, non‐vocational, systemic, and structural barriers still exist; hence, adults with a SPMI continue to face challenges with gaining and sustaining long‐term employment. Hence, it is important for these partnerships to be systematically set up to support the complexity of the employment journey for adults with a SPMI.

## INTRODUCTION

Serious persistent mental illness (SPMI) impacts different aspects of an individual’s life. This includes gaining and sustaining open, competitive employment and choice of work for adults with a SPMI (Devine *et al*. 2021; Gühne *et al*. 2021). It is well documented in evidence‐based studies across the globe that individuals with a mental illness experience higher levels of unemployment (Drake 2018; Modini *et al*. 2016) and occupational exclusion, thus hindering the type of work they would like to engage in (Devine *et al*. 2021; Gühne *et al*. 2021). However, individuals with a SPMI report a strong desire to work in competitive employment (Gühne *et al*. 2021; Petrakis *et al*. 2018), although they experience higher unemployment rates and many barriers that impact their vocational outcomes and choice of work (Bonfils *et al*. 2017; Hanisch *et al*. 2017).

Evidence‐based studies indicate that employment is one of the key factors in the recovery journey of individuals living with a mental illness (Drake 2018; Stirling *et al*. 2018). The benefits of and barriers to employment for individuals experiencing a mental illness have been widely researched and documented as well (Bonfils *et al*. 2017; Fernandez *et al*. 2017; Hanisch *et al*. 2017). Multiple studies on various vocational rehabilitation programmes for adults with a SPMI conducted globally focus more on social inclusion, mental well‐being, self‐esteem, and programme generalizability across countries and economies (Bejerholm *et al*. 2015; Bonfils *et al*. 2017; Drake 2018; Hoffmann *et al*. 2014; Modini *et al*. 2016; Waghorn *et al*. 2017, 2020). Supported employment (SE) models including the individual placement and support (IPS) model of SE along with various vocational rehabilitation approaches to assist individuals with a SPMI gain and sustain work have been widely researched, documented, and implemented with varying degrees of success overseas and across Australia (Bejerholm *et al*. 2015; Bonfils *et al*. 2017; Drake 2018; Hoffmann *et al*. 2014; McDowell *et al*. 2021; Modini *et al*. 2016; Waghorn *et al*. 2017). Vocational rehabilitation approaches based on the enhanced intersectoral links (EIL) approach and non‐IPS co‐location partnerships between adult community mental health teams (ACMHTs) and disability employment services (DES) have been implemented in very few ACMHTs across Australia (Chang *et al*. 2016; Petrakis *et al*. 2018; Stirling *et al*. 2018; Waghorn *et al*. 2020). However, there are limited studies and evidence‐based research on non‐IPS co‐location partnerships between ACMHTs and DES currently available.

Two Australian‐based studies by Devine *et al*. (2020a, 2020b) and Devine *et al*. (2021) explore; factors influencing employment outcomes and the notion of exercising consumer choice and control of DES participation and factors that promote sustainable work outcomes for individuals with a mental illness within the Australian DES context, respectively. A third Australian study published by Mellifont (2017) focussed on the need to reform the current Australian DES framework to assist participants with a mental illness gain and sustain employment. Devine *et al*. (2020a, 2020b) and Mellifont (2017) both highlight the need for improved integration and coordination of services between mental health and DES and greater consumer involvement to achieve employment outcomes for consumers with a SPMI.

The Australian DES system aims to assist individuals with a disability gain and sustain open employment by providing them with skills to prepare for, gain, and sustain employment in the long term as well as empowering them to choose and control the types of support they wish to receive while looking for employment (Department of Social Services [DSS] 2021). Although there is current research and evidence available within the Australian DES context, there are limited studies that focus on the relationship between the implementation of non‐IPS co‐location DES partnerships within ACMHTs and their impact on employment outcomes and consumer choice of work for adults with a SPMI. There is also limited evidence available to demonstrate the long‐term (greater than twelve months) impact of non‐IPS DES co‐location partnerships on employment outcomes for adults with a SPMI. This is an identified research and service gap that requires further exploration to gather evidence to facilitate and improve employment service provision for adults with a SPMI within the Australian mental health service and policy context. Hence, this systematic literature review undertaken by the authors aims to further explore the impact of non‐IPS co‐location partnerships between ACMHTs and DES, on employment outcomes and consumer choice of work for adults with a SPMI within the Australian mental health service and policy context. This paper will also highlight barriers to co‐location participation (Bonfils *et al*. 2017; Devine *et al*. 2021; Hanisch *et al*. 2017; McDowell *et al*. 2021; Stirling *et al*. 2018); develop guidelines to facilitate the implementation of non‐IPS co‐location partnerships across ACMHTs (Devine *et al*. 2021; Mellifont 2017; Waghorn *et al*. 2020); and provide policymakers and executive teams with evidence that will impact the implementation of co‐location partnerships across local health districts in NSW, in future (Aitken *et al*. 2020; Devine *et al*. 2021; Mellifont 2017; O’Leary 2005).

## METHODS

This systematic review aimed to investigate the impact of co‐location partnerships between ACMHS and DES on employment outcomes and consumer choice of work for adults with a SPMI. The Preferred Reporting Items for Systematic Reviews and Meta‐Analyses (PRISMA) methodology was used to conduct the systematic review (Page *et al*. 2021).

### Search strategy

A systematic literature search was conducted in CINAHL, Embase, MEDLINE, and PubMed from 01 January 2017 to 30 August 2021 to offer a review of the current literature on the subject. The initial search examined the relationship between mental health, employment, and DES using the Boolean operator ‘AND’. The first set of search terms was related to mental health: (‘mental health’ OR ‘mental illness’ OR ‘mental disorders’ OR ‘psychiatric illness’). The second set of search terms was related to employment: (‘employment’ OR ‘jobs’ OR ‘work’ OR ‘career’). The third set of search terms was related to DES: (‘disability employment services’). Only peer‐reviewed articles published in English were considered.

### Eligibility criteria

Inclusion criteria of the articles consisted of: (i) adults with a SPMI; (ii) peer‐reviewed journals; (iii) studies published between 01 January 2017 and 30 August 2021; (iv) employment services and outcomes; (v) job retention and sustainability; and (vi) Australian‐based studies. Exclusion criteria included populations with other health disorders, physical disabilities, and substance abuse as their primary diagnosis.

### Study selection

**Fig. 1:**
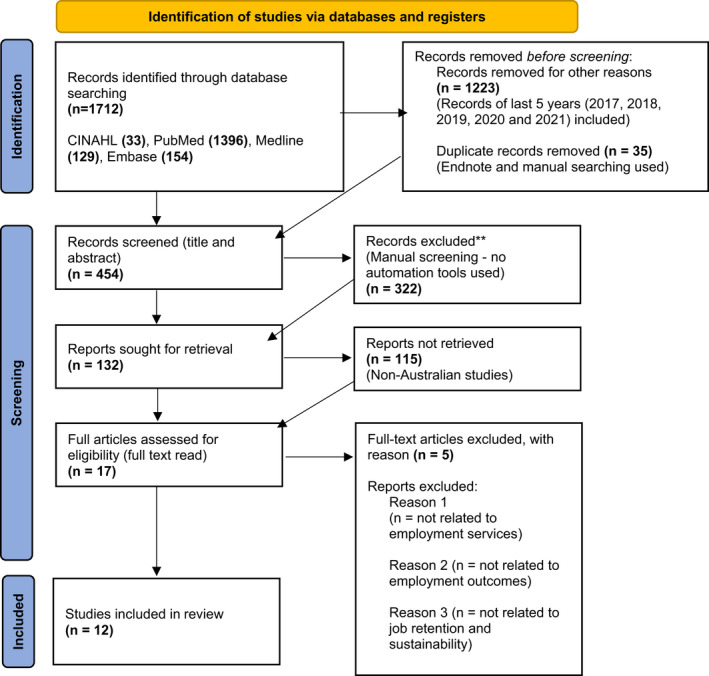
PRISMA flow diagram of the study selection process.

A total of 1712 articles were identified through database searching for this systematic review. All articles were imported into the EndNote literature manager software (version X9 for PC) and analysed using a thematic analysis. After removing duplicate articles (*n* = 35) and articles for ‘other’ reasons (*n* = 1223), 454 records were returned. The titles and abstracts of each article were manually screened by the author (no automation tools were used); this returned 132 articles. Non‐Australian‐based studies were excluded, which returned 17 articles out of which 12 met full inclusion criteria. The selection procedure of the articles is shown in Fig. 1.

### Quality analysis and data synthesis

The results of this review were performed using a narrative synthesis approach. The data were extracted according to the Cochrane manual (Higgins *et al*. 2011). Data were extracted by the first author from the included studies using a standardized data extraction form which was confirmed by the second author. The approach outlined by Harden *et al*. (2018) was adapted to assess the quality of the eligible studies identified. Literature was summarized and organized into specific categories and subcategories, which included authors, date of publications, study objectives, and methodology. The form also had a category for the findings, relevant additional information, and recommendations. A summary of the eleven included studies is outlined in Table 1, which contains study aims and objectives, main findings, and results and recommendations.

**Table 1:** Summary of included studies

Year	Authors	Study aim/Objectives	Research design/Methods	Main findings	Results & Recommendations
2020	Aitken *et al*.	To estimate the effect of disability acquisition on mental health	Quantitative: a longitudinal study	Disability acquisition was associated with a substantial decline in mental health, especially for those in low‐skilled jobs	Highlighted the need for social and health policies to reduce the mental health inequalities experienced by individuals who acquire a disability Suggested that policies focus on increasing employment rates, improving the sustainability of employment and providing employment services along with education and training for individuals who acquire a disability
2021	Devine *et al*.	To explore whether and how participants with psychosocial disability engaged with DES exercise choice and control to stay with or change DES providers	Qualitative: Narrative inquiry	No participant in this study exercised their choice to change DES providers despite dissatisfaction with the service Complex factors influence choice and control for DES participants	Suggested creating a DES system that addresses the life challenges and structural barriers faced by DES participants (with a psychosocial disability) more effectively
2020a	Devine *et al*.	To understand contextual factors that influence the engagement and potential benefits from DES programmes for DES participants with a psychosocial disability	Qualitative: Semi‐structured interview	Life challenges hinder DES participation and effectiveness for individuals with a mental illness	Despite ongoing reforms, the effectiveness of DES programmes for individuals with a psychosocial disability is undermined due to the life challenges experienced
2020b	Devine *et al*.	To explore factors influencing MH, well‐being, and personal recovery within the context of DES	Mixed methods: quantitative: survey; qualitative: in‐depth interviews	DES access to secure meaningful work Recovery‐oriented approach not consistently applied	Highlighted the importance of work in supporting the MH and well‐being of people with psychosocial disability Apply more consistent approach to DES practices
2017	Fernandez *et al*.	To create an integrated MH atlas of WSLHD to assist decision‐makers to plan services better based on local evidence	Mixed methods: quantitative: survey; qualitative: semi‐structured interview	Gaps in Australian MH care are a barrier to recovery The integrated MH Atlas of WSLHD identified 4 major gaps in MH care across WSLHD, one of which was the low availability of specific employment services for individuals with a lived experience of mental illness	Reforms considering current MH services should be developed The integrated MH atlas provides a tool for evidence‐informed planning and analysis of MH care across WSLHD Implications include using the integrated MH atlas to plan MH services better using local evidence
2021a	McDowell *et al*.	To further explore vocational service models and approaches to improve job tenure of individuals with a serious mental illness	Qualitative: narrative inquiry	Personal, organizational, and systemic issues make sustaining employment for individuals with a mental illness challenging	Several interventions outlined that could enhance job retention including family and peer support, as well as non‐clinical vocational approaches that can be adopted and implemented by service providers and employment specialists
2021b	McDowell *et al*.	To understand the views and practices that help and hinder employment specialists from DES while assisting individuals with a mental illness to gain employment	Qualitative: grounded theory; semi‐structured interview	DES employment specialists struggle with the pressure of meeting the needs of psychosocial clients while adhering to performance‐based finding and DES policies	Further training of DES staff is recommended to focus on client‐centred and recovery‐orientated services Systematic support for DES staff is recommended to adopt evidence‐based practices while working with the mentally ill
2017	Mellifont	To investigate the relationship between disability type (physical or psychiatric) and long‐term employment To identify evidence‐based measures that promote the long‐term employment of individuals with a mental illness engaged with DES	Qualitative: Narrative inquiry	Themes identified included resourcing, personalized support, and education Unemployment rates for individuals with a mental illness were almost 2.5 more than those with physical disabilities (over a 12‐month period)	Suggested a good practice employment guide based on identified themes Challenged Australian policymakers to ensure that funding exists for ongoing DES support for individuals with a mental illness beyond the initial 12‐month period Policy reform is required to improve the quality of employment services that are currently available to individuals with a mental illness
2017	Parletta *et al*.	To evaluate whether obligations to participate in supported employment were more beneficial to participants with a mental illness	Quantitative: cohort study	Motivation for participation in supported employment to gain employment is relatively independent of obligations, and hence counteracts beliefs among service providers that intrinsic motivation to gain employment is higher for participants with no obligations	Further investigations into participation in supported employment for participants receiving government payments versus participation of participants with no obligations linked to their government payments are required. Current government obligations can affect the delivery of supported employment that is evidence‐based
2018	Petrakis *et al*.	To assess whether IPS could be sustained if it was delivered by a joint partnership between AMH and employment services	Mixed methods: quantitative: survey; qualitative: in‐depth interviews	Employment was achieved by 46.3% of participants. This is one of two studies conducted that assessed the employment rate for adult MH consumers engaged in the IPS model of supported employment, in Australia	More trialling of supported employment programmes such as IPS, for MH consumers, is recommended, across all clinical services in Australia
2018	Stirling *et al*.	To explore challenges faced within the Australian context in implementing supported employment programmes such as IPS	Qualitative: document analysis	IPS principles are not adhered to by DES. Inadequate funding available to DES to implement supported employment programmes such as IPS	Policy refinement within the Australian DES context to better support individuals with a MH to gain and sustain employment. Better integration between employment and MH service delivery in the Australian policy and practice context
2020	Waghorn *et al*.	To summarize the major developments in Australia since the introduction of supported employment (IPS) in 2005 To outline the current situation and discuss future challenges and opportunities for individuals with a mental illness to obtaining and sustaining employment	Qualitative: document analysis	Supported employment expanded considerably within youth MH services. However, supported employment across ACMH services has been constrained	ACMH services to include vocational rehabilitation as part of individual recovery plans Policy changes are required to influence DES practice to become more evidence‐based as opposed to outcome‐based

Quality assessments were conducted to assess the accuracy of employment outcomes, consumer choice of work, client‐centred and recovery‐oriented service provision, and policies related to DES. Relevant Critical Appraisal Skills Programme (CASP) checklists guided the quality assessment of the qualitative literature as discussed by Long *et al*. (2020). The quality of the quantitative studies and the mixed‐methods studies were assessed using the Mixed Method Appraisal Tool (MMAT) (Hong *et al*. 2018). The quality assessment of the included studies was critically assessed by the second author using the above‐mentioned tools, which was verified by the first author. There was no disagreement between the reviewers for both tools and criteria. None of the selected studies were excluded based on the quality assessments results. The results of the quality appraisal show that all MMAT‐assessed studies met all criteria. For the studies assessed by the CASP, only two of the seven included studies did not meet one criterion each. The results of the quality assessments are presented in Table 2.

**Table 2:** Quality assessment Tools

Critical Appraisal Skills Program (CASP) for qualitative studies										
Study	Was there a clear statement of the aims of the research?	Is a qualitative methodology appropriate?	Was the research design appropriate to address the aims of the research?	Was the recruitment strategy appropriate to the aims of the research?	Were the data collected in a way that addressed the research issue?	Has the relationship between researcher and participants been adequately considered?	Have ethical issues been taken into consideration?	Was the data analysis sufficiently rigorous?	Is there a clear statement of findings?	Value of research
Devine *et al*. (2021)	Yes	Yes	Yes	Yes	Yes	Yes	Yes	Yes	Yes	Yes
Devine *et al*. (2020a)	Yes	Yes	Yes	Yes	Yes	Yes	Yes	Yes	Yes	Yes
McDowell *et al*. (2021a)	Yes	Yes	Yes	Yes	Yes	No	Yes	Yes	Yes	Yes
McDowell *et al*. (2021b)	Yes	Yes	Yes	Yes	Yes	Yes	Yes	Yes	Yes	Yes
Mellifont (2017)	Yes	Yes	Yes	Yes	Yes	No	Yes	Yes	Yes	Yes
Stirling *et al*. (2018)	Yes	Yes	Yes	Yes	Yes	Yes	Yes	Yes	Yes	Yes
Waghorn *et al*. (2020)	Yes	Yes	Yes	Yes	Yes	Yes	Yes	Yes	Yes	Yes

Mixed Methods Appraisal Tools (MMAT) for mixed‐methods studies							
Study	Are there clear research questions?	Do the collected data allow to address the research questions?	Is there an adequate rationale for using a mixed‐methods design to address the research question?	Are the different components of the study effectively integrated to answer the research question?	Are the outputs of the integration of qualitative and quantitative components adequately interpreted?	Are divergences and inconsistencies between quantitative and qualitative results adequately addressed?	Do the different components of the study adhere to the quality criteria of each tradition of the methods involved?
Devine *et al*. (2020b)	Yes	Yes	Yes	Yes	Yes	Yes	Yes
Fernandez *et al*. (2017)	Yes	Yes	Yes	Yes	Yes	Yes	Yes
Petrakis *et al*. (2018)	Yes	Yes	Yes	Yes	Yes	Yes	Yes

Mixed Methods Appraisal Tools (MMAT) for quantitative studies							
Study	Are there clear research questions?	Do the collected data allow to address the research questions?	Are the participants representative of the target population?	Are measurements appropriate regarding both the outcome and intervention (or exposure)?	Are there complete outcome data?	Are the confounders accounted for in the design and analysis?	During the study period, is the intervention administered (or exposure occurred) as intended?
Aitken *et al*. (2020)	Yes	Yes	Yes	Yes	Yes	Yes	Yes
Parletta *et al*. (2017)	Yes	Yes	Yes	Yes	Yes	Yes	Yes

## RESULTS

### Overview of studies

After reviewing the final twelve articles that fully met the inclusion criteria, the main characteristics of the studies are summarized in Table 1. All studies were Australian‐based and published between 01 January 2017 and 30 August 2021. This time frame was applied in order to make this review relevant to current policy and reforms in the context of the Australian DES system. Only peer‐reviewed journal articles were used to ensure credibility and reliability of information.

All studies focussed on the adult population as majority of paid employment is carried out during adult ‘working’ years generally between the ages of eighteen and sixty‐five, which was the focus of all studies. 42% of the studies used a qualitative approach; 25% used a mixed‐methods approach, while 17% of the studies used a quantitative approach. One study (8%) was a literature review, and one study (8%) was a document analysis. Structured interviews were used in almost half of the studies, thus making it the most common method of data collection. The majority of studies (60%) applied a thematic analysis, while 32% of the studies applied a descriptive (16%) and narrative (16%) analysis to represent information. Two studies (16%) were investigative in nature, and the only longitudinal study (8%) used a fixed‐effect linear regression model to capture information and report. The smallest number of participants used in the studies was ‘fourteen’ (*n* = 14), and the largest number of participants engaged was 2096 (*n* = 2096). All studies highlighted the impact of poor mental health and mental well‐being on gaining and sustaining employment for adults with a SPMI.

Three main themes and multiple subthemes emerged after the evaluation of the twelve included articles. These included the following: (i) IPS; (ii) DES practice, funding, policy, and reform within the Australian mental health system; and (iii) barriers to participation in DES programmes.

### IPS

Five studies focussed on IPS and its impact on vocational outcomes for individuals with a SPMI (McDowell *et al*. 2021; Parletta *et al*. 2017; Petrakis *et al*. 2018; Stirling *et al*. 2018; Waghorn *et al*. 2020). All studies indicated that IPS is an effective and successful model of SE for individuals with a SPMI. However, Waghorn *et al*. (2020) highlighted the fact that in Australia, the expansion of IPS across ACMHTs has been hindered as a result of the current Australian mental health system, its practices, and vocational service provision for adults with a SPMI. The study by Parletta *et al*. (2017) also highlighted that IPS has been a successful model of SE for individuals who are motivated to gain and sustain employment. Stirling *et al*. (2018) investigated reasons why IPS as a model of SE was not widely available throughout Australia.

#### Barriers to IPS implementation

Several barriers to IPS implementation have been highlighted by Parletta *et al*. (2017), Petrakis *et al*. (2018), Stirling *et al*. (2018), and Waghorn *et al*. (2020). These include a lack of understanding, education, and training on the implementation of IPS for mental health staff and DES providers; strict IPS implementation guidelines, which can be difficult to adhere to by mental health staff and DES providers; difficulty with achieving IPS fidelity scores, hence impacting the financial viability of IPS; and vocational rehabilitation not being acknowledged as a high priority and an integral part of an individual’s recovery journey (Parletta & Waghorn 2016; Parletta *et al*. 2017; Petrakis *et al*. 2018; Stirling *et al*. 2018; Waghorn *et al*. 2020).

### DES within the Australian mental health system

#### Practice

Several studies indicated the need for employment consultants (EC) from DES agencies, who work with adults with a SPMI, to be educated and trained with supporting participants to gain meaningful work and empower participants to exercise choice and control with their employment options and choice of DES providers (Devine *et al*. 2020a, 2020b, 2021). Studies also indicated that some DES providers undermined participant’s career aspirations and discouraged them from engaging in vocational activities such as further education and training. DES participants also reported negative experiences while engaged with a DES provider. These experiences reported included lack of provider knowledge and skill on how to support participants who have experienced past trauma; inability of providers to address participant’s diverse mental health needs; negative assumptions about participants; lack of respect shown towards participant’s circumstances and career goals; and lack of acknowledgement of participant’s employment history and current vocational capabilities (Devine *et al*. 2020a, 2020b, 2021; McDowell *et al*. 2021).

#### Funding

Studies indicated that the current DES funding system to support adults with a SPMI gain and sustain work is outcome‐focussed rather than individualized and recovery‐focussed (McDowell *et al*. 2021; Parletta *et al*. 2017; Waghorn *et al*. 2020). Waghorn *et al*. (2020) stated that the current system is funded by a complex model that includes a mix of federal and state funding, hence providing relatively short‐term support for mental health outcomes.

#### Policy and reform

All studies recommended changes to the current DES policies and reforms within the Australian mental health system. Studies indicated that current DES policies and reforms required reviewing and the implementation of specific changes in order to facilitate gaining and maintaining long‐term employment for adults with a SPMI.

### Barriers to participation in DES programmes

Seven studies highlighted the various barriers and challenges adults with a SPMI faced while attempting to engage and participate in DES, in order to gain and maintain paid employment. Studies indicated that significant barriers faced included fluctuations in mental health, inability to access employment services, poor education and training, and extended periods of unemployment. Life challenges such as inadequate and unstable housing, homelessness, financial insecurity, poor formal and informal support, disrupted education, and traumatic experiences all contributed to the barriers and challenges that adults with a SPMI faced while attempting to gain and maintain paid employment (Devine *et al*. 2020a, 2020b, 2021; Mellifont 2017; Petrakis *et al*. 2018; Stirling *et al*. 2018; Waghorn *et al*. 2020). Three studies acknowledged that adults with a SPMI experienced barriers to participation in DES programmes as a result of the health, social, and economic inequalities (Aitken *et al*. 2020; Devine *et al*. 2020a, 2020b). Other stated factors that contributed to barriers to participation included spending time in prison, discrimination faced as a result of receiving government payments, and being from non‐English‐speaking and refugee backgrounds. Overall, studies indicated that factors influencing mental health, well‐being, and personal recovery were all significant barriers that adults with a SPMI faced, which impacted their engagement and participation within the DES context (Devine *et al*. 2020a, 2020b, 2021; Mellifont 2017; Petrakis *et al*. 2018; Stirling *et al*. 2018).

## DISCUSSION

The aim of this systematic literature review was to investigate the impact of non‐IPS co‐location partnerships between ACMHTs and DES on employment outcomes and consumer choice of work for adults with a SPMI. During evaluation of included articles, it was found that studies focussed mainly on IPS, DES practice, funding, policy, and reforms within the Australian mental health system, benefits of employment, barriers to participation in SE programmes, and employment outcomes in relation to IPS. Only one study focussed on a ‘joint’ partnership between adult mental health services and employment services. However, this was in context of IPS implementation and service delivery. There was no literature that discussed consumer choice of work for adults with a SPMI nor investigated the impact of non‐IPS co‐location partnerships between ACMHTs and DES on employment outcomes for adults with a SPMI.

### Employment versus unemployment

Engagement in paid, open, competitive employment is often seen as a key factor in facilitating the personal and clinical recovery journey for adults with a SPMI (Aitken *et al*. 2020; Devine *et al*. 2020a, 2020b; Drake 2018; Stirling *et al*. 2018). Sustainable, long‐term employment ensures financial stability, enhances social inclusion and community integration, increases self‐confidence and self‐worth, instils a sense of personal achievement through contribution to society, reduces psychotic symptoms and relapses, and improves overall mental well‐being. It is also an important social and economic determinant of health. On the contrary, unemployment, job losses, and poor working conditions can lead to negative feelings such as feelings of hopelessness and decreased self‐esteem and can have detrimental effects on mental health and self‐efficacy while attempting to achieve future career goals (Aitken *et al*. 2020; Devine *et al*. 2020a, 2020b, 2021; McDowell *et al*. 2021).

Although the benefits of employment are glaringly obvious at an individual and community level for adults with a SPMI, the rate of unemployment for adults with a psychosocial disability is 38%, which is almost double the rate of unemployment for adults without a psychosocial disability (20%) (Australian Bureau of Statistics [ABS] 2021). Despite the Australian Government’s effort to reform the DES programmes, this gap in unemployment has been consistently increasing over the past twenty years or so (Devine *et al*. 2020a, 2020b, 2021; Mellifont 2017).

### IPS versus non‐IPS co‐location partnerships

Although IPS has been widely researched and praised for its successful vocational outcomes internationally (Bejerholm *et al*. 2015; Bonfils *et al*. 2017; Drake 2018; Hoffmann *et al*. 2014; Modini *et al*. 2016; Waghorn *et al*. 2017), its implementation across ACMHTs in Australia has been limited (Parletta *et al*. 2017; Petrakis *et al*. 2018; Stirling *et al*. 2018; Waghorn *et al*. 2020). Several factors such as the lack of education and training on the implementation of IPS and lack of application of IPS principles by DES providers have been blamed for its limited uptake. Other factors such as vocational rehabilitation not being acknowledged as high priority and part of individual recovery plans, and expressed concerns about the financial viability of IPS have been linked to the minimal implementation of IPS as a model of SE for adults with a SPMI (Parletta & Waghorn 2016; Parletta *et al*. 2017; Petrakis *et al*. 2018; Stirling *et al*. 2018; Waghorn *et al*. 2020).

Non‐IPS co‐location partnerships involve an EC from a local DES agency to physically co‐locate within an ACMHT. This enables adults with a SPMI wishing to gain paid employment register with the local DES agency in conjunction with the ACMHT. More recently, co‐location partnerships have been considered to be a viable vocational rehabilitation option due to the significant barriers that adults with a SPMI experience with DES participation (Chang *et al*. 2016; Devine *et al*. 2020a, 2020b, 2021; McDowell *et al*. 2021; Mellifont 2017; Petrakis *et al*. 2018; Stirling *et al*. 2018). Barriers faced affect DES engagement. International studies indicate that through co‐location partnerships, these barriers can be addressed and adults with a SPMI can be more supported with achieving their individual vocational goals (Drake 2018; Gühne *et al*. 2021; Hanisch *et al*. 2017; Modini *et al*. 2016).

### The Australian mental health system and DES

The current DES system is not integrated into the adult mental health system (Parletta *et al*. 2017; Waghorn *et al*. 2020). This lack of collaboration and formal agreement between mental health and employment services is a significant systemic barrier (Stirling *et al*. 2018). This impacts the inclusion of individuals with participation obligations and impacts a number of practices and services that are delivered to adults with a SPMI. Due to the mixed funding model across state and federal governments and systemic barriers, the current DES programmes are under‐resourced and inadequately funded and thus are incapable to address the ongoing issues related to non‐vocational barriers to employment (Devine *et al*. 2020a, 2020b, 2021). Some non‐vocational barriers experienced by adults with a SPMI include family breakdowns, poor physical health, adverse life events, and inadequate access to general and mental health services; all of which impact engagement and participation in DES programmes and the ability to gain and sustain long‐term employment for adults with a SPMI.

Fernandez *et al*. (2017) identified the low availability of specific employment services for individuals with a lived mental health experience and mental health diagnosis as one of the four major gaps in mental health care in Western Sydney Local Health District (WSLHD). This is of significance to the author as a vocational consultant across mental health services for WSLHD and the author’s involvement in various vocational rehabilitation programmes and projects for adults with a SPMI. Across WSLHD mental health services, currently there are no formal evidence‐based vocational rehabilitation programmes or co‐location partnerships between ACMHTs and DES to assist adults with a SPMI gain and sustain open, competitive employment. Although there are nine ACMHTs across the LHD, currently there are no information or data available on employment outcomes for adults with SPMI, whether engaged with DES or not.

### Recommendations

Based on current employment service provision, policies, reforms, and available literature, recommendations to support adults with a SPMI with gaining and sustaining long‐term employment within a non‐IPS model of SE include the following.

#### Addressing barriers and key challenges

Addressing barriers and key challenges such as vocational and non‐vocational barriers to work for adults with a SPMI include periods of unemployment, job retention, housing needs, stable accommodation, access to further education and training, physical and mental health, access to general and mental health services, and timely access to DES providers (Devine *et al*. 2020a, 2020b, 2021; McDowell *et al*. 2021). Addressing these key challenges will enhance long‐term employment outcomes, thus making employment sustainable for adults with a SPMI (McDowell *et al*. 2021; Modini *et al*. 2016).

Equipping DES staff with knowledge and skills to understand how the impact of ongoing mental health issues and past traumas influence employment and vocational goals through mental health education and training will enable them to address these barriers for adults with a SPMI (Devine *et al*. 2020a, 2020b). Providing mental health education and training for DES staff will enable them to support adults with a SPMI with gaining and sustaining employment rather than contributing to increased stress and trauma for participants. This will also lead to more appropriate job matching skills by DES staff for adults with a SPMI and equip staff with skills to promote self‐management within their participants (McDowell *et al*. 2021). Another benefit of providing mental health training and education is that DES staff will be able to better support participants through fluctuations in mental state, through periods of decreased motivation and ambivalence, and through life challenges (Chang *et al*. 2016). Linking job seekers with cognitive and psychological interventions, supported education and training, and ensuring that they have appropriate social supports, through either peer support workers, family, or friends, are other ways to address non‐vocational barriers to facilitate long‐term employment outcomes, thus making employment sustainable for adults with a SPMI (Devine *et al*. 2021; McDowell *et al*. 2021).

#### Addressing challenges related to the current DES programmes and system

Reviewing the current referral to and intake process of DES, matching participants with ECs that have the right skill, expertise, and capabilities including comprehensive knowledge of the current job market and have the ability to empathize with participants will contribute to higher levels of satisfaction and positive experiences for adults with a SPMI seeking employment through DES (Devine *et al*. 2021). Having a more coordinated and collaborative approach to supporting adults with a SPMI gain and sustain work through the DES system can be facilitated through integration of ACMH services and DES providers (Chang *et al*. 2016; Waghorn *et al*. 2017, 2020). Participants engaged in this process would be better supported with achieving their employment outcomes (Devine *et al*. 2021).

Promoting DES programmes through adult mental health services through co‐location partnerships; developing DES programmes that focus on client‐centred care as opposed to outcome‐based models; and addressing the issues of job satisfaction of DES staff to reduce the high turnover of staff currently experienced are some ways to address challenges faced by the current DES programmes and system (Devine *et al*. 2020a, 2020b, 2021; Hutchinson *et al*. 2018; Stirling *et al*. 2018). These will also decrease the pressure on DES providers to fulfil their current outcome‐based obligations and focus on providing a more client‐centred and continuously available employment service for adults with a SPMI (Bonfils *et al*. 2017; Hutchinson *et al*. 2018; Stirling *et al*. 2018; Waghorn *et al*. 2017).

#### Empowering consumers of DES and mental health services

Empowering consumers to exercise their choice and control of what they perceive would be best suited for them will ensure that they are part of the decision‐making process within their recovery journey (Devine *et al*. 2021). This empowerment will promote self‐belief and self‐worth. This will break the negative cycle of accepting jobs that are not suitable, thus leading to poor employment outcomes for adults with a SPMI. Often, DES participants believe that the ECs are ‘experts’ and feel obligated to accept decisions made by these ‘experts’. However, empowering consumers will enable them to speak up and share their opinions constructively so that they can exercise their right to choose their personal vocational pathway and shape their vocational journey. This empowerment will positively impact mental health, self‐esteem, and self‐confidence (Devine *et al*. 2021). Empowering consumers will also enable them to function optimally once discharge from ACMHTs while still engaged in DES programmes.

#### Addressing staff attitudes and beliefs towards mental health consumers wishing to work

Some evidenced‐based studies have described examples of DES participants’ experiences where their capacity to carry out employment‐related tasks has been undermined by DES and mental health staff (Devine *et al*. 2020a, 2020b; Hutchinson *et al*. 2018). Studies report that staff attitudes towards mental health consumers wishing to work have created self‐doubt and undermined their abilities relating to gaining employment. In such instances, participants have felt like they were ‘pushed’ into inappropriate or unsuitable jobs. Participants have reported that these experiences have caused them to lose their self‐confidence and have negatively impacted on their mental health and well‐being. Hence, addressing staff attitudes and beliefs towards mental health consumers wishing to work through education and training will facilitate the vocational journey for adults with a SPMI. Having a non‐judgemental attitude will also positively impact on their mental health and self‐confidence, thus fostering open and honest communication between staff and consumers wishing to gain and sustain open employment (Bonfils *et al*. 2017; Modini *et al*. 2016).

#### Addressing the availability of suitable employment

Devine *et al*. (2020a, 2020b) reported that the limited availability of suitable paid employment is a significant hindrance to improving vocational outcomes for adults with a SPMI. Job tenure, job satisfaction, and job retention are all impacted due to the limited availability of suitable paid employment for adults with a SPMI. Hence, job creation through alternative approaches to create new employment options to improve job tenure is an area that requires further investigation and development (McDowell *et al*. 2021). Developing and facilitating workplace accommodations, as well as providing specific training to adults with a SPMI seeking employment, could possibly become another approach to creating more suitable jobs and workplace environments. This can only assist with prompting job satisfaction, enhancing job retention, and increasing job tenure. Supporting employers to build a diverse workforce with a variety of skills, experiences, and capabilities would not only create more suitable jobs but would also minimize discrimination and exclusion experienced by adults with a SPMI when entering the workforce (Drake 2018).

#### Addressing policy reforms

Although the Australian Government has been committed to making sure that all individuals with disabilities have choice and control of how they receive employment services, adults with a SPMI frequently fall through these service gaps (Devine *et al*. 2021; Stirling *et al*. 2018). One of the main reasons for this occurrence is they often experience increased exclusion and discrimination across different areas of life when compared to individuals experiencing non‐psychosocial disability (Department of Social Services [DSS] 2021; Devine *et al*. 2021). New DES policy and reform will become available from 2022 (DSS 2021). These reforms focus on new employment service models with a strong focus on supporting youth via the New Employment Service Model (Department of Education, Skills & Employment [DESE] 2021). Siloed policies and reforms such as these highlight some of the reasons why adults with a SPMI fall through service gaps (Devine *et al*. 2021).

An effective and functional DES system has to be shaped by clearly stated reforms and policies that addresses vocational, non‐vocational, and structural barriers faced by adults with a SPMI wishing to gain and sustain employment. Addressing structural barriers through policy refinement will improve mental well‐being, vocational outcomes, and overall life situations for adults with a SPMI (Devine *et al*. 2021; Modini *et al*. 2016).

Parletta *et al*. (2017) state that a policy shift with the DES system is required to ensure that DES can provide evidenced‐based employment services for adults with a SPMI. Policymakers should be challenged with developing policies that aim for good DES practices with a shift from outcome‐orientated to recovery‐oriented, evidenced‐based, individualized, and integrated employment services for adults with a SPMI (Devine *et al*. 2020a, 2020b; Stirling *et al*. 2018; Waghorn *et al*. 2020). Reviewing and modifying current DES policies to decrease the stress on ECs assist adults with a SPMI gain and sustain work so that they can offer tailored pathways to address participant’s needs (McDowell *et al*. 2021). Providing practical solutions through policy reform such as decreasing EC caseloads so that they can provide a more intensive service to a fewer participants would help them to better support participants with their vocational needs. This will reduce the current result‐focussed DES practices (Waghorn *et al*. 2020). Policy change could provide incentives for DES providers who can support their participants for longer periods of time – greater than the current requirements of two years. This will ensure that adults with a SPMI are suitably matched according to their abilities and will retain their jobs for longer periods of time. Strategic policy reforms such as these will enhance the quality of employment services currently received by adults with a SPMI (Mellifont 2017; Stirling *et al*. 2018).

### Implications for clinical practice

Mental health clinicians are skilled with working with adults with a SPMI as they are trained to support them with their symptoms, medication, managing their overall mental health, and general well‐being. DES staff are skilled with supporting individuals with a range of disabilities to gain employment (Stirling *et al*. 2018; Waghorn *et al*. 2020). Hence, to improve vocational outcomes for adults with a SPMI, it is highly recommended that clinical mental health services and DES collaborate more and become better integrated. Co‐location partnerships between ACMHTs and DES are one way to increase collaboration and ensure integration of the two services that support adults with a SPMI gain and sustain long‐term employment. To establish, facilitate, implement, and maintain successful co‐location partnerships between DES and ACMHTs, the following guidelines are recommended. These guidelines have been influenced and developed by reviewing the twelve chosen studies and studies by Bejerholm *et al*. (2015); Bonfils *et al*. (2017); Chang *et al*. (2016); Drake (2018); Hoffman *et al*. (2014); Hutchinson *et al*. (2018); Modini *et al*. (2016); Parletta & Waghorn 2016; and Waghorn *et al*. (2017), in order to facilitate implementation of co‐location partnerships between DES and ACMHTs.

### Guidelines

#### Inter‐service collaboration

A memorandum of understanding (MOU) to be drawn up between mental health services and DES to enable an EC from a local DES provider to attend the corresponding local ACMHT once a week, for four hours, will outline the responsibilities and functions of the ACMHT and DES provider in order to facilitate this partnership. Both parties have to be agreeable to the MOU. A vocational champion from the ACMHT has to be appointed to coordinate and facilitate the partnership. Joint education and training of mental health and DES staff on co‐location partnerships has to be occurred prior to drawing up the MOU.

#### Consumer‐centred, individualized, and recovery‐oriented model of care

The EC will attend the second half of the morning handover meeting to give an opportunity to mental health clinicians to discuss potential consumer referrals and gain feedback from current referrals. The EC will be available to mental health staff and consumers for the remainder of the four hours, as they will be physically present (co‐locating) with the ACMHT. The EC and the mental health clinicians will work together with consumers interested in engaging with the DES. Clinicians are skilled at providing ongoing care for the mental health needs of consumers, while ECs support them with their vocational goals. Clinicians have to ensure that their consumer’s vocational goals are recorded in their care and wellness plans. These plans are to be monitored, reviewed, and updated at regular intervals by clinicians.

#### Timely service provision

The EC will complete the registration for the consumer to be linked in with the DES provider. Once this occurs, the job‐seeking process will commence. The EC will conduct a career and skills assessment to identify the consumer’s perceived abilities and strengths. This will assist the consumer to identify the next steps (along with the EC and mental health clinician), towards gaining employment. Depending on the results of the career and skills assessment, the consumer may have to engage in further education and/or training. The EC will then assist the consumer to pursue this so that they will be able to look for suitable employment upon completion of their further education and/or training. Consumers may commence looking for suitable employment after accessing the results of their career and skills assessment. The consumer will be supported by the EC and mental health clinician during their job‐seeking process.

#### Increased flexibility

Consumers will have a choice of what day, time, and week they would like to attend appointments with the EC co‐locating within the ACMHT. Consumers will be informed of available times and dates prior to making appointments. This allows consumers to schedule appointments with the EC, and their mental health clinician and/or their psychiatrist on the same day or different days/alternate weeks.

#### The job‐seeking process

While continuing to have regular contact with mental health staff on ACMHTs, consumers will be supported by the EC through the job‐seeking process. This can include preparing a resume, practising interviewing skills, learning telephone skills, and how to operate digital switchboards. Consumers will also be supported with job applications and interviews, as well as appropriate job‐related attire via the DES providers. Other consumer needs during this process will be identified, discussed, and appropriately met, where possible.

#### Reporting and feedback

Verbal reporting and feedback has to be occurred weekly at the morning handover meeting. Monthly reports need to be provided by the EC, and specific data relating to the job‐seeking process need to be collected. This will be recorded on a spreadsheet to be shared with the ACMHT.

#### Ongoing support and monitoring

Consumers engaged with co‐locations partnerships will be regularly supported for a period of two years as per the current DES policy guidelines. Many consumers are discharged from ACMHS at some stage during their two years of engagement with co‐location partnerships. Hence, two years post‐engagement with DES and ACMHS, most consumers do not receive ongoing support from either agency. Unless there is policy reform, ongoing support for consumers will have to be provided by non‐government organizations and community mental health agencies after two years of DES engagement.

### Future research and planning

Implementation of co‐location partnerships across local health districts in NSW should provide policymakers and executive teams with evidence and direction for future planning of employment services for adults with a SPMI. It is also recommended that further research is conducted to identify the ongoing needs and support required by employed adults with a SPMI beyond the current two‐year obligation of DES support received. Further research should be carried out to ascertain what is required by adults with a SPMI to retain and sustain employment beyond two years of support from DES. Mental health and DES policymakers should collaboratively address challenges, barriers, and structural and systemic issues outlined to develop cohesive and integrated policies that will positively impact and enhance employment service delivery for adults with a SPMI. This means that current state government‐funded adult mental health services have to collaborate and integrate with federal government‐funded employment services.

## CONCLUSION

The employment journey for adults with a SPMI is complex. However, this should be a part of their recovery journey. Various models of SE to assist adults with a SPMI gain and sustain employment have been tried and tested with varying degrees of success across Australia and internationally. The current Australian DES system provides employment support for adults with a SPMI. Although the Australian Government has made efforts to ensure that adults with a SPMI have better access to employment services, gaps in services and siloed policies still prevail. These are unfavourable for adults with a SPMI looking for work and sustaining work in the long term. Vocational, non‐vocational, systemic, and structural barriers still exist; hence, adults with a SPMI continue to face challenges with gaining and sustaining long‐term employment. The author proposes the implementation of co‐location partnerships between ACMHTs and DES providers to enhance and facilitate suitable long‐term sustainable employment for adults with a SPMI. Using a systematic literature review and other evidence‐based studies, the author has developed guidelines for the implementation of co‐location partnerships between ACMHTs and DES providers to improve employment outcomes for adults with a SPMI.

## ACKNOWLEDGEMENT

Open access funding provided by University of New England.

## References

[inm13007-bib-0001] Aitken, Z. , Simpson, J. A. , Bentley, R. , Milner, A. , LaMontagne, A. D. & Kavanagh, A. M. (2020). Does the effect of disability acquisition on mental health differ by employment characteristics? A longitudinal fixed‐effects analysis. Social Psychiatry and Psychiatric Epidemiology, 55 (8), 1031–1039. 31650207 10.1007/s00127-019-01783-x PMC7395044

[inm13007-bib-0002] Australian Bureau of Statistics (2021). Mental health. Available from: URL: https://www.abs.gov.au/statistics/health/health‐conditions‐and‐risks/mental‐health/latest‐release

[inm13007-bib-0003] Bejerholm, U. , Areberg, C. , Hofgren, C. , Sandlund, M. & Rinaldi, M. (2015). Individual placement and support in Sweden—A randomized controlled trial. Nordic Journal of Psychiatry, 69 (1), 57–66. 10.3109/08039488.2014.929739 24983382

[inm13007-bib-0004] Bonfils, I. S. , Hansen, H. , Dalum, H. S. & Eplov, L. F. (2017). Implementation of the individual placement and support approach – facilitators and barriers. Scandinavian Journal of Disability Research, 19 (4), 318–333. 10.1080/15017419.2016.1222306

[inm13007-bib-0005] Chang, L. , Douglas, N. , Scanlan, J. N. & Still, M. (2016). Implementation of the enhanced intersectoral links approach to support increased employment outcomes for consumers of a large metropolitan mental health service. British Journal of Occupational Therapy, 79 (11), 643–650.

[inm13007-bib-0006] Department of Education, Skills and Employment (2021). New employment services model. Available from: URL: https://www.dese.gov.au/new‐employment‐services‐model

[inm13007-bib-0007] Department of Social Services (2021). Disability employment services. Available from: URL: https://www.dss.gov.au/our‐responsibilities/disability‐and‐carers/programmes‐services/disability‐employment‐services

[inm13007-bib-0008] Devine, A. , Dickinson, H. , Brophy, L. , Kavanagh, A. & Vaughan, C. (2021). ‘I don’t think they trust the choices I will make’. – Narrative analysis of choice and control for people with psychosocial disability within reform of the Australian disability employment services program. Public Management Review, 23 (1), 10–30. 10.1080/14719037.2019.1648700

[inm13007-bib-0009] Devine, A. , Vaughan, C. & Kavanagh, A. (2020). If I had stable housing I would be a bit more receptive to having a job. Factors influencing the effectiveness of disability employment services reform. Work, 65 (4), 775–787. 32310208 10.3233/WOR-203130

[inm13007-bib-0010] Devine, A. , Vaughan, C. , Kavanagh, A. *et al*. (2020). ‘I’m proud of how far I’ve come. I’m just ready to work’: Mental health recovery narratives within the context of Australia’s disability employment services. BMC Public Health, 20, 325. 10.1186/s12889-020-8452-z 32164650 PMC7068916

[inm13007-bib-0011] Drake, R. E. (2018). Employment and schizophrenia: Three innovative research approaches. Schizophrenia Bulletin, 44 (1), 20–21. 10.1093/schbul/sbx170

[inm13007-bib-0012] Fernandez, A. , Gillespie, J. A. , Smith‐Merry, J. *et al*. (2017). Integrated mental health atlas of the Western Sydney Local Health District: Gaps and recommendations. Australian Health Review, 41 (1), 38–44. 10.1071/AH15154 27007640

[inm13007-bib-0013] Gühne, U. , Pabst, A. , Löbner, M. *et al*. (2021). Employment status and desire for work in severe mental illness: Results from an observational, cross‐sectional study. Social Psychiatry and Psychiatric Epidemiology, 56 (9), 1657–1667. 33860804 10.1007/s00127-021-02088-8 PMC8429146

[inm13007-bib-0014] Hanisch, S. E. , Wrynne, C. & Weigl, M. (2017). Perceived and actual barriers to work for people with mental illness. Journal of Vocational Rehabilitation, 46 (1), 19–30.

[inm13007-bib-0015] Harden, A. , Thomas, J. , Cargo, M. *et al*. (2018). Cochrane qualitative and implementation methods group guidance series—paper 5: Methods for integrating qualitative and implementation evidence within intervention effectiveness reviews. Journal of Clinical Epidemiology, 97, 70–78. 10.1016/j.jclinepi.2017.11.029 29242095

[inm13007-bib-0016] Higgins, J. P. , Thomas, J. & Chandler, J. (Eds). (2011). Cochrane Handbook for Systematic Reviews of Interventions. Hoboken, NJ: John Wiley & Sons.

[inm13007-bib-0017] Hoffmann, H. , Jäckel, D. , Glauser, S. , Mueser, K. & Kupper, Z. (2014). Long‐term effectiveness of supported employment: 5‐year follow‐up of a randomized controlled trial. American Journal of Psychiatry, 171 (11), 1183–1190. 25124692 10.1176/appi.ajp.2014.13070857

[inm13007-bib-0018] Hong, Q. N. , Fàbregues, S. , Bartlett, G. *et al*. (2018). The Mixed Methods Appraisal Tool (MMAT) version 2018 for information professionals and researchers. Education for Information, 34, 285–291. 10.3233/EFI-180221

[inm13007-bib-0019] Hutchinson, J. , Gilbert, D. , Papworth, R. & Boardman, J. (2018). Implementing supported employment. Lessons from the *Making IPS Work Project* . International Journal of Environmental Research and Public Health, 15 (7), 1545. 10.3390/ijerph15071545 PMC6069163 30037092

[inm13007-bib-0020] Long, H. A. , French, D. P. & Brooks, J. M. (2020). Optimising the value of the critical appraisal skills programme (CASP) tool for quality appraisal in qualitative evidence synthesis. Research Methods in Medicine & Health Sciences, 1 (1), 31–42. 10.1177/2632084320947559

[inm13007-bib-0021] McDowell, C. , Ennals, P. & Fossey, E. (2021). Vocational service models and approaches to improve job tenure of people with severe and enduring mental illness: A narrative review. Frontiers in Psychiatry, 12, 1116. 10.3389/fpsyt.2021.668716 PMC8298859 34305676

[inm13007-bib-0022] McDowell, C. , Fossey, E. & Harvey, C. (2021). Moving clients forward: A grounded theory of disability employment specialists’ views and practices. Disability and Rehabilitation, 1–9. Advance online publication. 10.1080/09638288.2021.1937341 34190004

[inm13007-bib-0023] Mellifont, D. (2017). DESperately seeking service: A narrative review informing a disability employment services reform framework for Australians with mental illness. Work (Reading, Mass.), 58 (4), 463–472. 10.3233/WOR-172643 29254128

[inm13007-bib-0024] Modini, M. , Tan, L. , Brinchmann, B. *et al*. (2016). Supported employment for people with severe mental illness: Systematic review and meta‐analysis of the international evidence. British Journal of Psychiatry, 209 (1), 14–22. 10.1192/bjp.bp.115.165092 27103678

[inm13007-bib-0025] O’Leary, Z. (2005). Researching Real‐World Problems: A Guide to Methods of Inquiry. London, UK: Sage.

[inm13007-bib-0026] Page, M. J. , McKenzie, J. E. , Bossuyt, P. M. *et al*. (2021). The PRISMA 2020 statement: An updated guideline for reporting systematic reviews. BMJ, 372, n71. 10.1136/bmj.n71 PMC8005924 33782057

[inm13007-bib-0027] Parletta, V. A. & Waghorn, G. (2016). The financial viability of evidence‐based supported employment for people with mental illnesses in a blended funding system. Journal of Vocational Rehabilitation, 44 (2), 227–241.

[inm13007-bib-0028] Parletta, V. A. , Waghorn, G. & Dias, S. (2017). The applicability of supported employment to adults with participation obligations as a condition for receiving welfare benefits. American Journal of Psychiatric Rehabilitation, 20 (2), 106–125.

[inm13007-bib-0029] Petrakis, M. , Stirling, Y. & Higgins, K. (2018). Vocational support in mental health service delivery in Australia. Scandinavian Journal of Occupational Therapy, 26 (7), 535–545. 10.1080/11038128.2018.1498918 30301392

[inm13007-bib-0030] Stirling, Y. , Higgins, K. & Petrakis, M. (2018). Challenges in implementing individual placement and support in the Australian mental health service and policy context. Australian Health Review, 42 (1), 82–88. 28104039 10.1071/AH16093

[inm13007-bib-0031] Waghorn, G. , Killackey, E. , Dickson, P. , Brock, L. & Skate, C. (2020). Evidence‐based supported employment for people with psychiatric disabilities in Australia: Progress in the past 15 years. Psychiatric Rehabilitation Journal, 43 (1), 32. 31204823 10.1037/prj0000370

[inm13007-bib-0032] Waghorn, G. , Logan, J. , Dias, S. & Hielscher, E. (2017). Vocational functioning among people with psychiatric disabilities five to seven years after receiving supported employment services. Journal of Rehabilitation, 83 (2), 36–42.

